# Perceptions of Use of Names, Recognition of Roles, and Teamwork After Labeling Surgical Caps

**DOI:** 10.1001/jamanetworkopen.2023.41182

**Published:** 2023-11-17

**Authors:** Becky J. Wong, Aussama K. Nassar, Michelle Earley, Ling Chen, Teresa Roman-Micek, Samuel H. Wald, Tait D. Shanafelt, Sara N. Goldhaber-Fiebert

**Affiliations:** 1 Department of Anesthesiology, Perioperative and Pain Medicine, Stanford University School of Medicine, Stanford, California; 2 Department of Surgery, Stanford University School of Medicine, Stanford, California; 3 Department of Surgery, Division of General Surgery, Stanford School of Medicine, Stanford, California; 4 Interventional Platform Education, Stanford, California; 5 Interventional Platform Education, Stanford, California; 6 Division of Hematology, Department of Medicine, Stanford School of Medicine, Stanford, California

## Abstract

**Importance:**

Communication failures in perioperative areas are common and have negative outcomes for both patients and clinicians. Names and roles of teammates are difficult to remember or discern contributing to suboptimal communication, yet the utility of labeled surgical caps with names and roles for enhancing perceived teamwork and connection is not well studied.

**Objective:**

To evaluate the use of labeled surgical caps in name use and role recognition, as well as teamwork and connection, among interprofessional perioperative teammates.

**Design, Setting, and Participants:**

In this quality improvement study, caps labeled with names and roles were distributed to 967 interprofessional perioperative clinicians, along with preimplementation and 6-month postimplementation surveys. Conducted between July 8, 2021, and June 25, 2022, at a single large, academic, quaternary health care center in the US, the study comprised surgeons, anesthesiologists, trainees, and all interprofessional hospital staff who work in adult general surgery perioperative areas.

**Intervention:**

Labeled surgical caps were offered cost-free, although not mandatory, to each interested clinician.

**Main Outcome and Measure:**

Quantitative survey of self-reported frequency for name use and role recognition as well as postimplementation sense of teamwork and connection. The surveys also elicited free response comments.

**Results:**

Of the 1483 eligible perioperative clinicians, 967 (65%; 387 physicians and 580 nonphysician staff; 58% female) completed preimplementation surveys and received labeled caps, and 243 of these individuals (51% of physicians and 8% of staff) completed postimplementation surveys. Pre-post results were limited to physicians, due to the low postsurvey staff response rate. The odds of participants reporting that they were often called by their name increased after receiving a labeled cap (adjusted odds ratio [AOR], 13.37; 95% CI, 8.18-21.86). On postsurveys, participants reported that caps with names and roles substantially improved teamwork (80%) and connection (79%) with teammates. Participants who reported an increased frequency of being called by their name had higher odds for reporting improved teamwork (AOR, 3.46; 95% CI, 1.91-6.26) and connection with teammates (AOR, 3.21; 95% CI, 1.76-5.84). Free response comments supported the quantitative data that labeled caps facilitated knowing teammates’ names and roles and fostered a climate of wellness, teamwork, inclusion, and patient safety.

**Conclusions and Relevance:**

The findings of this quality improvement study performed with interprofessional teammates suggest that organizationally sponsored labeled surgical caps was associated with improved teamwork, indicated by increased name use and role recognition in perioperative areas.

## Key Points

**Question:**

Is use of labeled surgical caps associated with improved name use and role recognition, as well as teamwork and connection for perioperative clinicians?

**Findings:**

In this quality improvement study using preintervention and postintervention surveys, use of labeled surgical caps was associated with participants being referred to by their name and recognized by their role; use of caps was also associated with improved sense of teamwork and connection. Those who experienced an improvement in being called by their name reported the most positive outcomes regarding teamwork and connection with teammates.

**Meaning:**

The findings of this study suggest that organization-sponsored use of labeled surgical caps is a relatively low-cost mechanism to facilitate use of name, role identity, and teamwork among perioperative teams.

## Teaser

This quality improvement study assesses frequency of name use and role recognition among perioperative teams before and after the use of caps labeled with individuals' names and roles.

## Introduction

Use of names fosters team communication and function, helping both clinicians and patients.^1,2,3,4^ Previous studies found that communication failures in the operating room (OR) occur in a third of team exchanges^3^ contributing to inefficiency, team tension, and procedural errors.^5^ The Surgical Time Out includes team members stating their names and roles to enable effective communication.^6^ Yet, names and roles are difficult to remember^7,8,9,10^ and there is often a lack of familiarity of OR teammates’ names and roles between different specialties (eg, surgeons and anesthesiologists), especially in large centers where teammates rotate.^10^ The COVID-19 pandemic expanded the use of masks and goggles outside the OR, making it challenging to recognize teammates.^11^ Increased reliance on temporary workers to address staffing shortages introduced a large and persistent number of unfamiliar teammates.^12,13^ Patients also frequently struggle to identify the names and roles of clinicians on their care team, with few able to name their physicians.^14^ Notably, patient satisfaction is associated with the number of physicians that the patient can identify.^15^

Name and role identification can foster clinician diversity, equity, inclusion, and well-being, especially for women and underrepresented minority populations who are often mistaken for nonphysician roles.^16,17,18,19,20^ Underrepresented minority clinicians are frequently mistaken for others of the same race,^21^ and implicit gender bias contributes to women being perceived as less competent in intellectual fields.^22^ Such stereotypes and role misidentification cause feelings of exclusion, anger, and decreased work satisfaction.^23,24^

One proposed intervention is to have clinicians’ roles more prominently displayed on their badges. Studies suggest that having the word “doctor” in large print on physician badges improves role identification by patients and other team members as well as professional satisfaction, especially among women physicians.^25,26,27^ Studies have explored related approaches to foster name and role identity in the perioperative areas, with one suggesting 94% of perioperative clinicians would support widespread adoption of name and role labels on caps.^28^ In 2018, a social media campaign, #TheatreCapChallenge, was led by an Australian anesthetist.^29^ This sparked enthusiasm for the concept^30^ and multiple small, short-term, and/or single-discipline studies suggested positive outcomes, such as labeled caps for cesarean delivery teams increasing name use and decreasing missed communications,^4^ resident anesthesiologists being addressed more often by name by the surgical teams,^31^ and improved teamwork and communication within smaller groups in ORs.^32^

Systematic institution-level efforts are needed to translate these approaches to routine practice and evaluate their broad outcomes. To our knowledge, no large interprofessional studies of the use of labeled caps in perioperative areas have been reported to date. We hypothesized that offering interprofessional perioperative clinicians thoughtfully designed, labeled surgical caps with names and roles would increase the likelihood of being referred to by name, foster role identity, and thereby enhance perceived teamwork and connection.

## Methods

We followed the Standards for Quality Improvement Reporting Excellence (SQUIRE) 2.0 reporting guideline. This interprofessional study of labeled surgical caps was conducted from July 8, 2021, to June 25, 2022. Survey data collection occurred from July 8 to December 31, 2021, for preintervention and March 22 to June 25, 2022, for postintervention. Stanford Healthcare’s Quality Improvement review determined that this was not human participant research and was exempt from further institutional review board review, with no patients involved, no consent forms required, and all participants’ data deidentified before analyses. Race and ethnicity and gender data were gathered to establish whether certain groups benefited differentially from labeled caps. The human resources department provided deidentified information on gender and rank. Race and ethnicity was self-reported in the preintervention survey, and Other was an option if no specific category fit, with no write-in fields. All participants were emailed the choice of opting out of deidentified information on gender and rank. All data were stored in the university’s password-protected, encrypted servers. An overview of the methods is given in the eMethods in Supplement 1.

### Participants

Eligible participants in perioperative areas were surgeons, anesthesiologists, trainees, nurses, technicians, and other nonphysician staff (subsequently referred to herein as staff). Identified clinicians were emailed a voluntary, cost-free order form with brief survey questions. There were announcements at faculty and staff meetings and Quick Response code posters visible in relevant perioperative areas. Completion of the presurvey was required to receive labeled caps.

### Intervention

Labeled cloth surgical caps were designed for clear legibility: solid color fabrics with embroidered name and role in relatively large, clear font and high-contrast lettering on the forehead area. Name wording for each role was standardized based on focus group input. For example, physicians requested Dr first-name last-name. Staff requested listing only first names. Preferred first names were allowed (eg, nicknames or middle names). Role labels were standardized for clarity, eg, resident surgeon or RN, based on focus group feedback on many possibilities and tradeoffs between teammate role clarity, patient understanding of terminology, consistency, and preferences (eFigure 1 in Supplement 1).

Given that personal style emerged as an important factor of clinician adoption, color and style of cap as well as comfort were carefully considered. Diverse options were provided to support preferences with 13 colors and 4 styles offered to accommodate head sizes and hair volumes. Caps were purchased from a US cottage industry business that allowed for iterative design input. Embroidered cap costs were within the market range of $25 to $45.

Depending on the department funding, 2 to 4 caps were provided to each participant to allow for alternating and laundering. Cap ordering and wearing were voluntary. The surgical Time Out continued to include name introductions and participants continued wearing their standard identification name badges.

### Assessment

Four of the 6 postintervention survey questions regarding names and roles were identical to the presurvey questions, with a 5-point Likert scale asking participants (1) how often teammates called them by their name, (2) how often teammates mistook their role, (3) how well the responder knew teammates’ names, and (5) how well the responder knew teammates’ roles (eAppendix in Supplement 1). The preintervention survey also asked about discomfort talking to teammates whose name or role they could not remember.

Postsurveys were administered to participants approximately 6 months after receiving their caps to give them time to experience the use of labeled caps. The postintervention survey included 2 additional items exploring whether labeled caps had improved teamwork (as a proxy for outcome regarding patient care) and connection with teammates (as a proxy for outcome regarding participant well-being). In the postsurvey, respondents were also asked for free-text comments regarding their experience wearing labeled caps. These qualitative comments (eFigure 4 and eFigure 5 in Supplement 1) were grouped by thematic area, although without formal qualitative analyses given scope and resource limitations.

Responses to quantitative survey questions were dichotomized into often (responses of always or often) and not often (responses of sometimes, rarely, or never). A reverse scoring approach was used for the question, “How often did others mistake your role?,” with rarely or never (favorable) classified as not often and sometimes, always, or often (unfavorable) classified as often. This approach preserved the conceptual consistency evaluating the proportion of responders indicating the 2 most favorable options on the Likert scale.

### Statistical Analysis

Due to low postintervention survey response rate among staff, pre-post analyses were limited to physicians. Primary outcomes were self-reported name use and role recognition, as well as perceived outcome regarding use of labeled caps with teamwork and connection. The McNemar test was used to evaluate the change in proportion of physicians reporting correct name and role recognition before and after receiving a labeled cap. Physicians who completed both the presurvey and postsurvey were included for this paired test.

Multivariable mixed-effects logistic regression models with a random intercept were also used to estimate the association between receiving a labeled surgical cap and each of the name use and role recognition outcomes. Differential improvement was evaluated through interaction terms and stratified models. Physicians who completed at least 1 survey were included for these models. Ordinal logistic regression models were used to evaluate factors associated with a better sense of teamwork and connection after receiving a labeled cap and were run for physicians who completed both presurveys and postsurveys. All models were adjusted for gender, race and ethnicity, rank, and role. For mixed-effects logistic and ordinal logistic regression models, any demographic subgroup with less than 10 participants (eg, for some gender categories) was not analyzed separately to avoid overfitting. As a sensitivity analysis, we also performed all of the above analyses on the entire study population, including both staff and physicians for both presurveys and postsurveys. A 2-tailed *P* < .05 was considered statistically significant. All analyses were conducted using SAS, version 9.4 (SAS Institute Inc).

## Results

Of the 1483 eligible perioperative teammates invited to participate, 967 (65%) individuals (58% female; 42% male) completed the preintervention survey and received labeled caps (387 physicians and 580 staff). Participant demographic and professional characteristics are reported in Table 1. Among the 967 participants, 243 (51% of physicians and 8% of staff) also answered the postsurvey (eFigure 2 in Supplement 1). The gender and race and ethnicity of physicians who completed both the presurvey and postsurvey were similar to physicians who completed only the presurvey (eTable in Supplement 1).

**Table 1.** Demographic Characteristics^a^

Characteristic	Total, No. (%)
Participants	967
Gender	
Female	539 (58)
Male	386 (42)
Not declared	7 (1)
Nonbinary	1 (<1)
Missing	34
Race and ethnicity^b^	
American Indian or Alaska Native	7 (1)
Asian	377 (40)
Black or African American	61 (6)
Hispanic or Latino	72 (8)
Native Hawaiian or other Pacific Islander	29 (3)
White	307 (32)
Other	96 (10)
Missing	18
Physician/staff	
Physician	387 (40)
Staff	580 (60)
Role	
Resident anesthesiologist	80 (8)
Resident surgeon	103 (11)
Fellow anesthesiologist	3 (<1)
Fellow surgeon	20 (2)
Attending anesthesiologist	102 (11)
Attending surgeon	75 (8)
Nurse	312 (32)
Technician	202 (21)
Other^c^	66 (7)
Missing	4

^a^ Categories listed as how they appear in the survey.

^b^ Subgroups with less than 10 participants were not analyzed separately to avoid overfitting.

^c^ Self-selected option.

**Figure 1.**
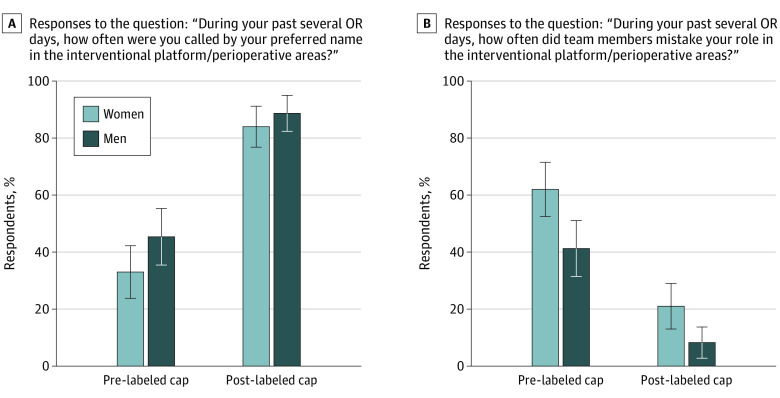
Response to Questions on Changes in Name and Role Recognition by Gender Response to questions on how often the respondents were called by name (A) and whether their role was mistaken (B) following use of labeled caps. OR indicates operating room. Error bars indicate 95% CIs.

Preintervention, most participants (622 of 936 [66%]) agreed or strongly agreed with the statement, “I feel uncomfortable talking to other teammates when I cannot remember their names or roles” (eFigure 3 in Supplement 1). Paired data were available for 197 physicians who completed both the preintervention and postintervention surveys. Physicians reported a significant improvement in being often called by their name after receiving a labeled cap. This improvement was observed both in the frequency of being often called by name (preintervention: 39%; 95% CI, 32%-46%; postintervention: 86%; 95% CI, 81%-91%; *P* < .001) (Figure 1A) and increased odds of being called by name from multivariable models (adjusted odds ratio [AOR], 13.37; 95% CI, 8.18-21.86) (Table 2).

**Table 2.** Improvements in Name Use and Role Recognition^a^

Outcome	Post–labeled cap vs pre–labeled cap, AOR (95% CI)^b^^,^^c^
Called by name	13.37 (8.18-21.86)
Role mistaken^d^	0.17 (0.11-0.26)
Know names of others	4.52 (3.05-6.71)
Know roles of others	2.33 (1.62-3.35)

Abbreviation: AOR, adjusted odds ratio.

^a^ Results include physician participants who took at least a pre or post survey (n = 383).

^b^ All models were adjusted for race and ethnicity, role, rank, and gender.

^c^ All findings significant at *P* < .001.

^d^ Note different directionality, ie, an improvement is less mistaken role.

Survey data also indicated a significant reduction in mistaken role after receiving a labeled cap, evident in both the frequency of mistaken role (preintervention: 52%; 95% CI, 45%-59%; postintervention: 15%; 95% CI, 10%-20%; *P* < .001) (Figure 1B) and reduced odds of mistaken role from multivariable models (AOR, 0.17; 95% CI, 0.11-0.26) (Table 2).

There were also improvements in the frequency of knowing the names and roles of teammates (eFigure 6 and eFigure 7 in Supplement 1), with consistent results seen in multivariable models (Table 2). Absolute improvements in name use and role recognition from preintervention to postintervention were similar across gender, race and ethnicity, rank, and role.

**Figure 2.**
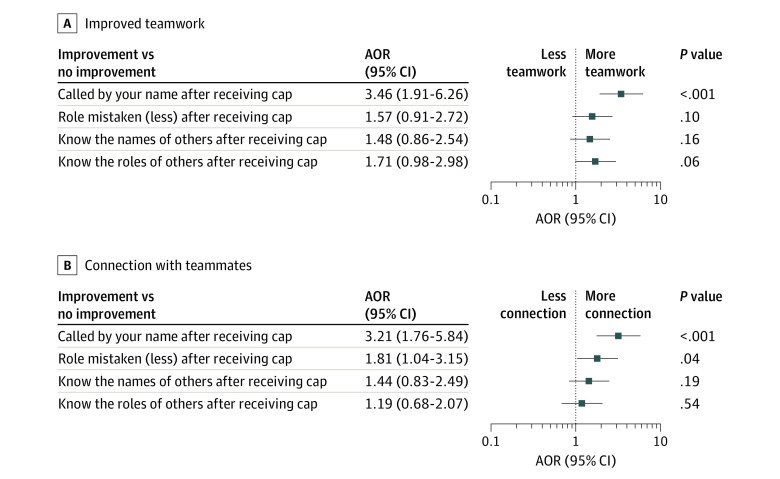
Response to Questions on Changes in Teamwork and Connection With Team Members Response to questions on whether the respondents believed teamwork (A) and connection to teammates (B) was changed following use of labeled caps. AOR indicates adjusted odds ratio. Error bars indicate 95% CIs.

For teamwork, there was a sense of substantial improvement, rated as quite a bit or very much (80%) and for connection (79%) after cap implementation. Moreover, physicians who reported being called more often by their name had higher odds for reporting a sense of improved teamwork (AOR, 3.46; 95% CI, 1.91-6.26; *P* < .001) and of connection with teammates (AOR, 3.21; 95% CI, 1.76-5.84; *P* < .001) (Figure 2).

## Discussion

The results of this quality improvement study found that, despite widespread use of name badges, baseline name use and role recognition among perioperative teammates were poor: less than half of participants reported that they were often called by their name and most often had their role mistaken. Among physicians, significant improvements were observed after the introduction of labeled caps. Physicians also perceived improved teamwork and connection with teammates after the use of labeled caps, especially among those reporting being called by their name more frequently. Collectively, these results suggest that organization-sponsored use of labeled surgical caps is a useful, relatively low-cost intervention to facilitate name use, role identity, teamwork, and connection among perioperative teammates. Our results corroborate earlier studies reporting that being called by name is associated with perceived teamwork and sense of connection, which are known substrates for patient safety and clinician well-being.^32,33^

These study findings also have implications for equity and inclusion. Consistent with earlier studies,^18,19,25,26,27,34^ baseline use of name and role recognition were less favorable for women and individuals with fewer years of training. Although implementing labeled caps did not eliminate the disparities in the outcomes measured, the magnitude of improvement in use of names and correct identification of roles with the introduction of labeled surgical caps among women was larger than the baseline disparity between men and women. The same was found for trainees vs attendings. There was no baseline difference in name use for individuals in underrepresented minorities compared with those not part of underrepresented minorities, but there was a disparity in correct identification of roles. While introducing labeled caps will not solve complex sociocultural issues, it appears to be a simple and pragmatic intervention with meaningful impacts. One participant commented, “[As] a woman of color, the caps have been extremely affirming for my role as a fellow surgeon in the OR. It reduces the cognitive fatigue and underlying anticipation of being mistaken for a different role (often resident, med student, or nurse).” Since vulnerable subgroups often start at worse baseline levels and derived similar absolute improvements, and since there can be a threshold effect for feeling valued, labeled caps may be particularly beneficial for these populations.

Our study showed that faculty attendings were 3 times more likely than trainees (residents/fellows) to be called by their name. This may be due to attendings’ more stable presence in a given work area and seniority. Surgeons were almost 4 times more likely than anesthesiologists to be called by their name, supporting a previous study reporting that surgeons were more often correctly named than others in the OR.^9^ While trainees and anesthesiologists (and their patients and teams) may benefit most from labeled caps, wearing labeled caps by seniors and leaders encourages others to do so and was powerful in our campaign. Qualitative comments also suggested surgeons valued labeled caps that enabled them to recall names of perioperative teammates with whom they infrequently work.

A study of anesthesiology residents wearing labeled caps increased OR personnel and surgical team knowing the anesthesiology resident’s name.^31^ An interprofessional perioperative personnel study with 236 presurveys and 107 postsurveys found that responders reported knowing names of all OR members more frequently, but, contrary to our study findings, did not find a statistically significant increase in teamwork.^32^ That study evaluated the use of labeled caps over a short interval (2 weeks) and may have been underpowered to detect moderate results. Another study suggested that the use of labeled caps to highlight names and roles and facilitate intraoperative teamwork helped clinicians who have less common names.^35^

Our finding that most participants felt uncomfortable communicating with teammates when unable to remember their names or roles represents an important baseline barrier to perioperative communication. After implementation of labeled caps, there was a sense of improved teamwork and connection, especially among those who were called by their names more often. Likely contributing factors include decreasing barriers to communicating with teammates,^36^ increased engagement with name use,^37^ and hearing others’ communication in noisy ORs when prefaced by one’s own name (ie, the cocktail party effect).^38,39^ Using an individual’s name has been reported to decrease apprehension when asking for clarification,^40^ produce faster response time,^41^ and increase the ability to shift one’s focus more reliably.^42^ Labeled caps to facilitate closed-loop communication using names is a practical and cost-effective strategy to improve communication.^43^

There has been historical debate on the perceived infection risk of surgical caps. Multiple independent studies reported no increase in surgical site infection risk based on type and style of surgical caps.^44,45,46,47^ Based on these data, a convening of experts from surgery, nursing, and anesthesiology associations^48,49,50^ issued a formal statement in 2019 asserting that type of head coverings was not associated with surgical site infections.^51^ In fact, banning cloth caps might have negative consequences on communication, team dynamics, and patient safety given the difficulty of identifying names.^52^ Despite the evidence and expert position statements, there remain institutions with policies banning cloth caps. For these institutions, alternative approaches to harness the benefits of labeled caps might include printing names on labels, writing names on adhesive tape to be used on disposable caps, or considering a policy change in light of the published evidence.

### Limitations

This study has limitations. Data on how presurvey nonresponders differed from participants were not available. However, the presurvey needs assessment results remain of interest given reasonably high response rates for all clinician groups. For physicians, postintervention survey nonresponders were similar to responders in the demographic variables assessed. Given the low staff postsurvey response rates, pre-post data analyses were limited to physicians. Potential contributors to low staff response rates postintervention include temporary staff (eg, a quarter of the nursing staff was temporary), overall staff turnover, lack of paid time to complete surveys for hourly employees, and a nurse strike during the postintervention survey period. In addition, the generalizability of our single-institution study to other institutions and contexts is unknown. For example, small practices/groups in which teammates do not change or rotate and most teammates are familiar may find limited relevance. It is possible, however, that even in these settings labeled caps may help patients identify the name and roles of members of their health care team. As for any innovation, the implementation process affects adoption and behavioral change.^53^ Additional studies are needed to further evaluate the impact of labeled surgical caps in diverse practice settings.

To our knowledge, this is the largest study evaluating use of labeled caps for interprofessional teams in perioperative areas and builds on the findings of earlier studies with multiple statistically significant and substantial impacts. Participants were progressively enlisted over 5 months, with a potential for those enrolling later to have seen the labeled caps of earlier participants, possibly influencing the study. To evaluate the impact of this variable, the time from study start to preintervention survey completion was factored in the regression models and did not significantly alter the results.

## Conclusions

In this single-institution quality improvement study, distribution of standardized, organization-sponsored surgical caps labeled with name and role increased the odds of clinicians being called by name, fostered role identity, and enhanced perceptions of teamwork and connection among perioperative teams, all of which may also improve patient safety. The data from this study indicate that the utility of simple and pragmatic interventions to enhance inclusion, teamwork, and well-being should not be underestimated.

## Notes

Supplement 1. **eMethods.** Overview **eFigure 1.** Labeled Caps **eAppendix.** Pre- and Post-Survey Questions **eFigure 2.** Study Flow Diagram **eTable.** Demographics by Responder Status for Physicians **eFigure 3.** Uncomfortable Not Knowing Names and Roles **eFigure 4.** Name-Relevant Post-Intervention Participant Comments **eFigure 5.** Role-Relevant Post-Intervention Participant Comments **eFigure 6.** Know Names of Others **eFigure 7.** Know Roles of Others

Supplement 2.
Data Sharing Statement
