# Supplementary material for: Perceptions of Use of Names, Recognition of Roles, and Teamwork After Labeling Surgical Caps
Source: JAMA Netw Open. 2023 Nov 17;6(11):e2341182. doi: 10.1001/jamanetworkopen.2023.41182 (PMC10656635; doi:10.1001/jamanetworkopen.2023.41182)
Supplement: Supplement 1. — eMethods. Overview eFigure 1. Labeled Caps eAppendix. Pre- and Post-Survey Questions eFigure 2. Study Flow Diagram eTable. Demographics by Responder Status for Physicians eFigure 3. Uncomfortable Not Knowing Names and Roles eFigure 4. Name-Relevant Post-Intervention Participant Comments eFigure 5. Role-Relevant Post-Intervention Participant Comments eFigure 6. Know Names of Others eFigure 7. Know Roles of Others [file jamanetwopen-e2341182-s001.pdf]

Supplementary Online Content

Wong BJ, Nassar AK, Earley M, et al. Perceptions of use of names, recognition of roles, and teamwork after labeling surgical caps. *JAMA Netw Open*. 2023;6(11):e2341182. doi:10.1001/jamanetworkopen.2023.41182

eMethods. Overview

eFigure 1. Labeled Caps

eAppendix. Pre- and Post-Survey Questions

eFigure 2. Study Flow Diagram

eTable. Demographics by Responder Status for Physicians

eFigure 3. Uncomfortable Not Knowing Names and Roles

eFigure 4. Name-Relevant Post-Intervention Participant Comments

eFigure 5. Role-Relevant Post-Intervention Participant Comments

eFigure 6. Know Names of Others

eFigure 7. Know Roles of Others

This supplementary material has been provided by the authors to give readers additional information about their work.

© 2023 Wong BJ et al. *JAMA Network Open.*

eMethods. Overview

Figures include paired data only (participants who took both the pre-survey and post-survey). Models include physician participants who took at least one survey. Covariates from multivariable models control for confounding and also provide information on differences across gender, race, and role that are not related to the intervention of receiving a labeled cap.

© 2023 Wong BJ et al. *JAMA Network Open.*

eFigure 1. Labeled caps

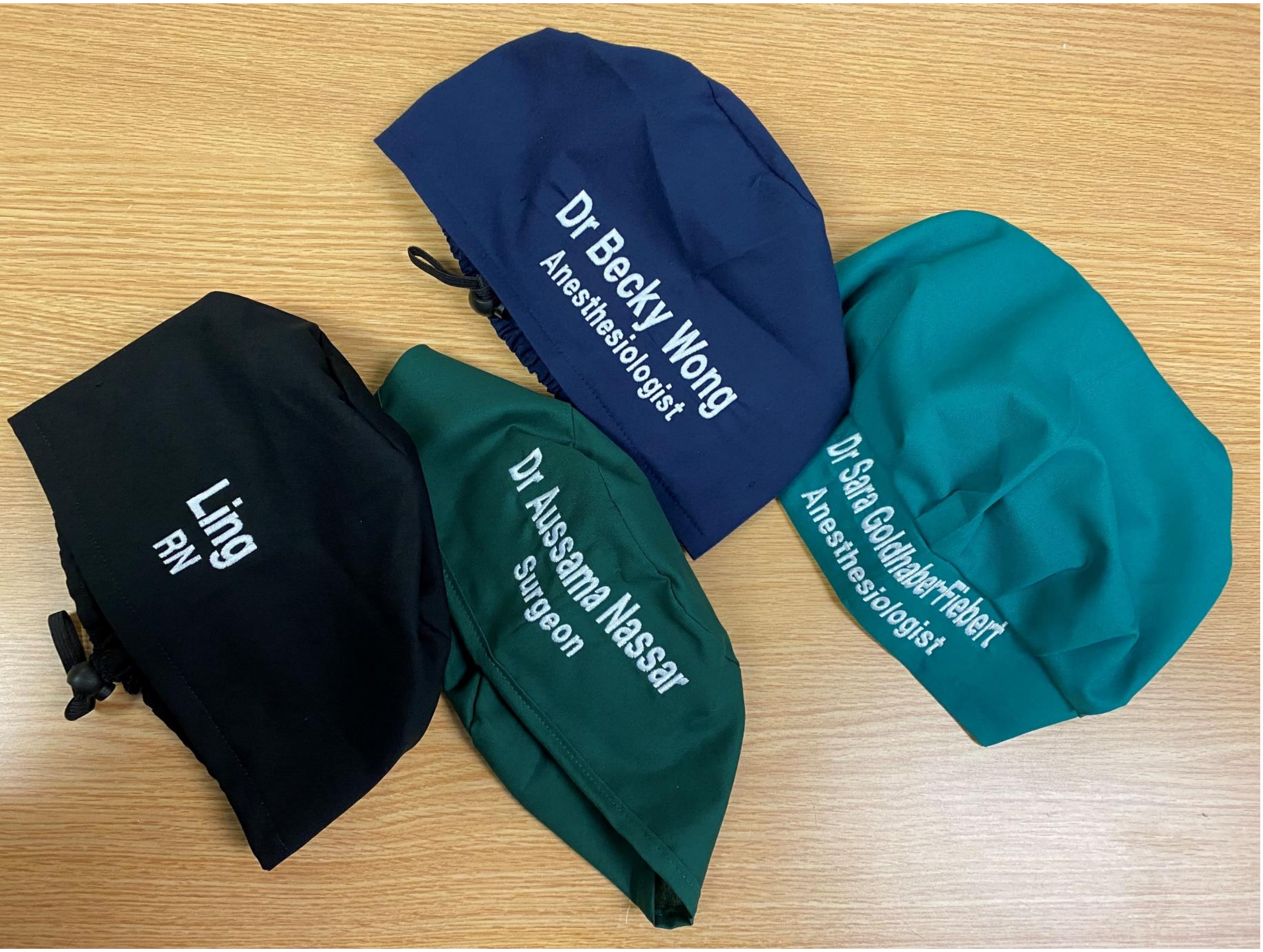

© 2023 Wong BJ et al. *JAMA Network Open.*

eAppendix. Pre- and post-survey questions

**Pre-Survey Questions**
During your past several OR days, how often were you called by your PREFERRED NAME in the Interventional Platform/Perioperative areas?<br><br>Always, Often, Sometimes, Rarely, Never
During your past several OR days, how often did team members MISTAKE YOUR ROLE, in the Interventional Platform/Perioperative areas?<br><br>Always, Often, Sometimes, Rarely, Never
When I see team members in the Interventional Platform/Perioperative areas, I know their NAMES.<br><br>Always, Often, Sometimes, Rarely, Never
When I see team members in the Interventional Platform/Perioperative areas, I know their ROLES<br><br>Always, Often, Sometimes, Rarely, Never
I feel uncomfortable talking to other team members when I CANNOT remember their NAMES or ROLES.<br><br>Strongly agree, Agree, Neither agree nor disagree, Disagree, Strongly Disagree

© 2023 Wong BJ et al. *JAMA Network Open.*

Self-identified Ethnicity/Race<br><br>We ask this question to understand how diversity, equity, and inclusion issues impact the use of names in the Interventional Platform/Perioperative areas. For example, unfamiliar names may be difficult to pronounce.<br><br>American Indian or Alaska Native<br><br>Asian<br><br>Black or African American<br><br>Hispanic or Latino<br><br>Native Hawaiian or Other Pacific Islander<br><br>White<br><br>Other
Self-Identified Gender (deidentified, and via Human Resources database, with participants able to opt-out):<br><br>Female<br><br>Male<br><br>Non-Binary<br><br>Not Declared
**Post-Survey Questions**
During your past several OR days, how often were you called by your PREFERRED NAME in the Interventional Platform/Perioperative areas?<br><br>Always, Often, Sometimes, Rarely, Never

© 2023 Wong BJ et al. *JAMA Network Open.*

During your past several OR days, how often did team members MISTAKE YOUR ROLE, in the Interventional Platform/Perioperative areas?<br><br>Always, Often, Sometimes, Rarely, Never
When I see team members in the Interventional Platform/Perioperative areas, I know their NAMES.<br><br>Always, Often, Sometimes, Rarely, Never
When I see team members in the Interventional Platform/Perioperative areas, I know their ROLES<br><br>Always, Often, Sometimes, Rarely, Never
Caps with names and roles have IMPROVED TEAMWORK in the Interventional Platform/Perioperative areas.<br><br>Very much, Quite a bit, Moderately, A little bit, Not at all
Caps with names and roles have INCREASED MY CONNECTION with team members in the Interventional Platform/Perioperative areas.<br><br>Very much, Quite a bit, Moderately, A little bit, Not at all
Please share your experiences with the caps--positive or negative. <Free response text>

© 2023 Wong BJ et al. *JAMA Network Open.*

eFigure 2. Study flow diagram

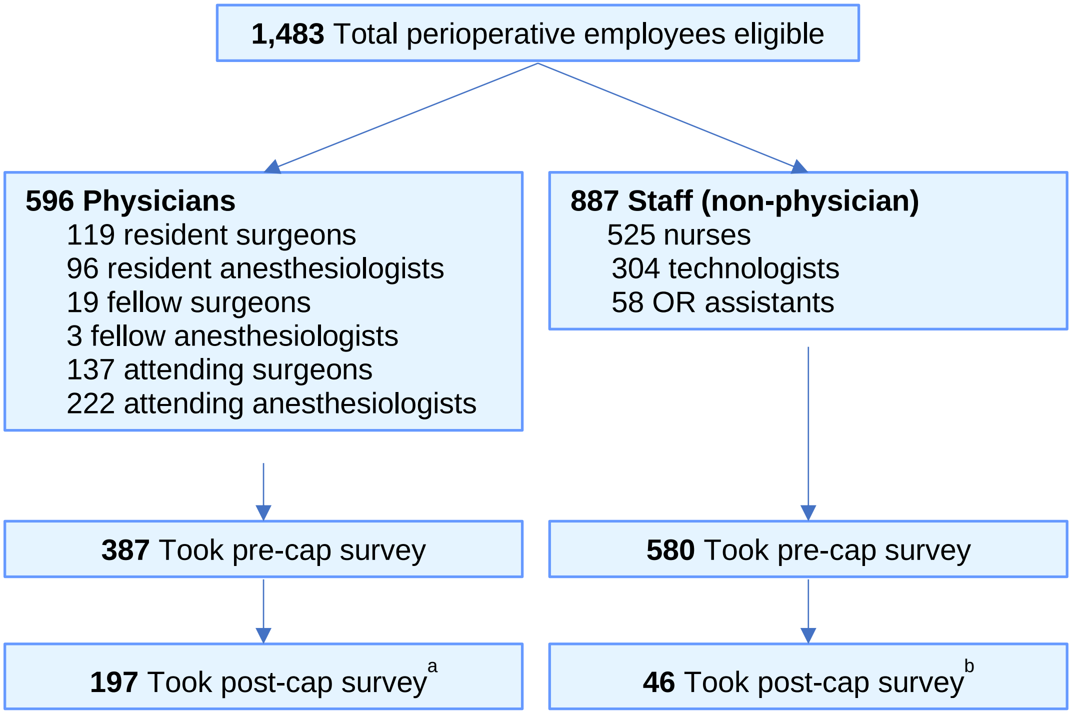

[a,b]*14 physicians, 17 staff took only the post-survey because they were away for the pre-survey*

© 2023 Wong BJ et al. *JAMA Network Open.*

eTable. Demographics by responder status for physicians

		Took pre- and post-survey Yes (N=197)	No (N=190)	Total (N=387)	P-Value[a]
Sex	Female	100 (51%)	85 (45%)	185 (48%)	0.24
	Male	97 (49%)	105 (55%)	202 (52%)	
Underrepresented minority	Yes	33 (17%)	41 (22%)	74 (19%)	0.22
	No	164 (83%)	148 (78%)	312 (81%)	
	Missing	0	1	1	

[a]Chi-Square Test

© 2023 Wong BJ et al. *JAMA Network Open.*

eFigure 3. Uncomfortable not knowing names and roles

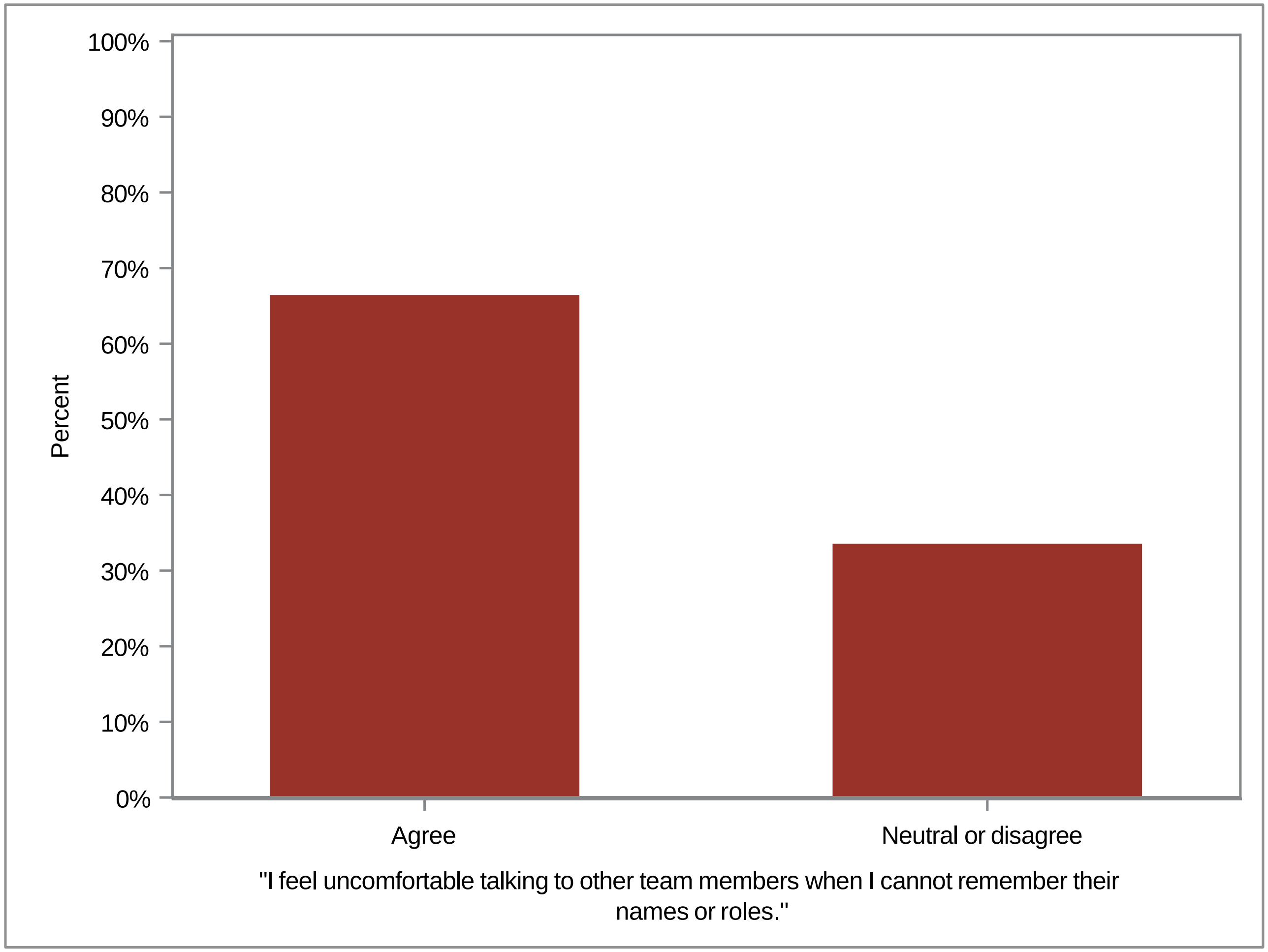

*Bars represent answers of agree (*agree *or* strongly agree*) and neutral or disagree (*neither agree nor disagree, disagree*, or* strongly disagree*)* (N=936)*. Note that 967 took a pre-survey, but 31 participants did not answer this specific question.*

© 2023 Wong BJ et al. *JAMA Network Open.*

eFigure 4. Name-relevant post-intervention participant comments

"I've never had an issue being called "anesthesia" by the surgery or nursing teams. I recognize it's not ideal, but it didn't bother me. So it was a huge surprise to me how uplifting it feels to be called by my name by the rest of the perioperative team. It truly does make me feel a stronger team connection and more engaged with the rest of the OR."

-male anesthesiology resident

"[By wearing the labeled caps] I have a name in a place where I can lose my identity and worth especially as a trainee"

-female surgery fellow

"Since I have an uncommon name, I used to spell it or show my badge when I introduce myself. The cap shows my name clearly, I don't even have to say it, staff members already know who I am and patients too."

-female RN

© 2023 Wong BJ et al. *JAMA Network Open.*

eFigure 5. Role-relevant post-intervention participant comments

"[As] a woman of color, the caps have been extremely affirming for my role as a fellow surgeon in the OR. It reduces the cognitive fatigue and underlying anticipation of being mistaken for a different role (often resident, med student or nurse). I also feel more confident in addressing my anesthesia colleagues by name- often I want to convey my respect, but it's hard for me to learn names right away."

-female surgery fellow

"Patients sometimes will look [at the labeled cap] and know that they are being seen by a doctor."

-female surgery resident

"I have found them incredibly helpful. They allow me to assert my role as a leader in the OR more easily and in a manner that improves interaction with other team members…. I wish everyone would wear them at all times to facilitate communication."

-female attending surgeon

"As a young female physician, there are multiple ways that the caps have improved my experience at work. I am correctly identified as a physician and in charge of my case more often"

- female attending anesthesiologist

"It's been great to be able to walk into a room and have people know who I am and what my role is."

-male anesthesiology resident

© 2023 Wong BJ et al. *JAMA Network Open.*

eFigure 6. Know names of others

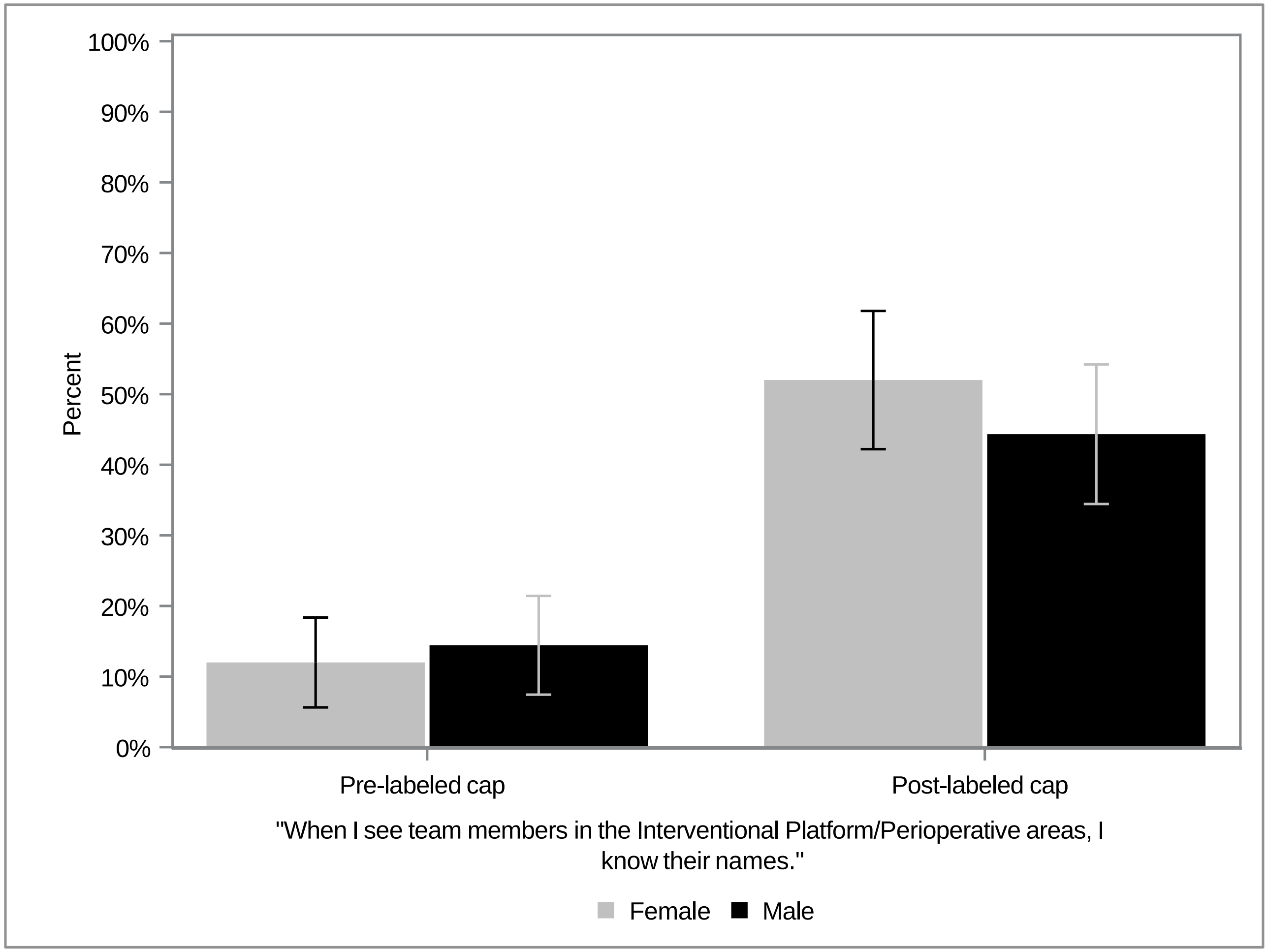

Bars represent answers of always *or* often *(vs.* sometimes*,* rarely*, or* never*) and are from paired responses for physicians only (N = 197). A higher percentage is favorable.*

© 2023 Wong BJ et al. *JAMA Network Open.*

eFigure 7. Know roles of others

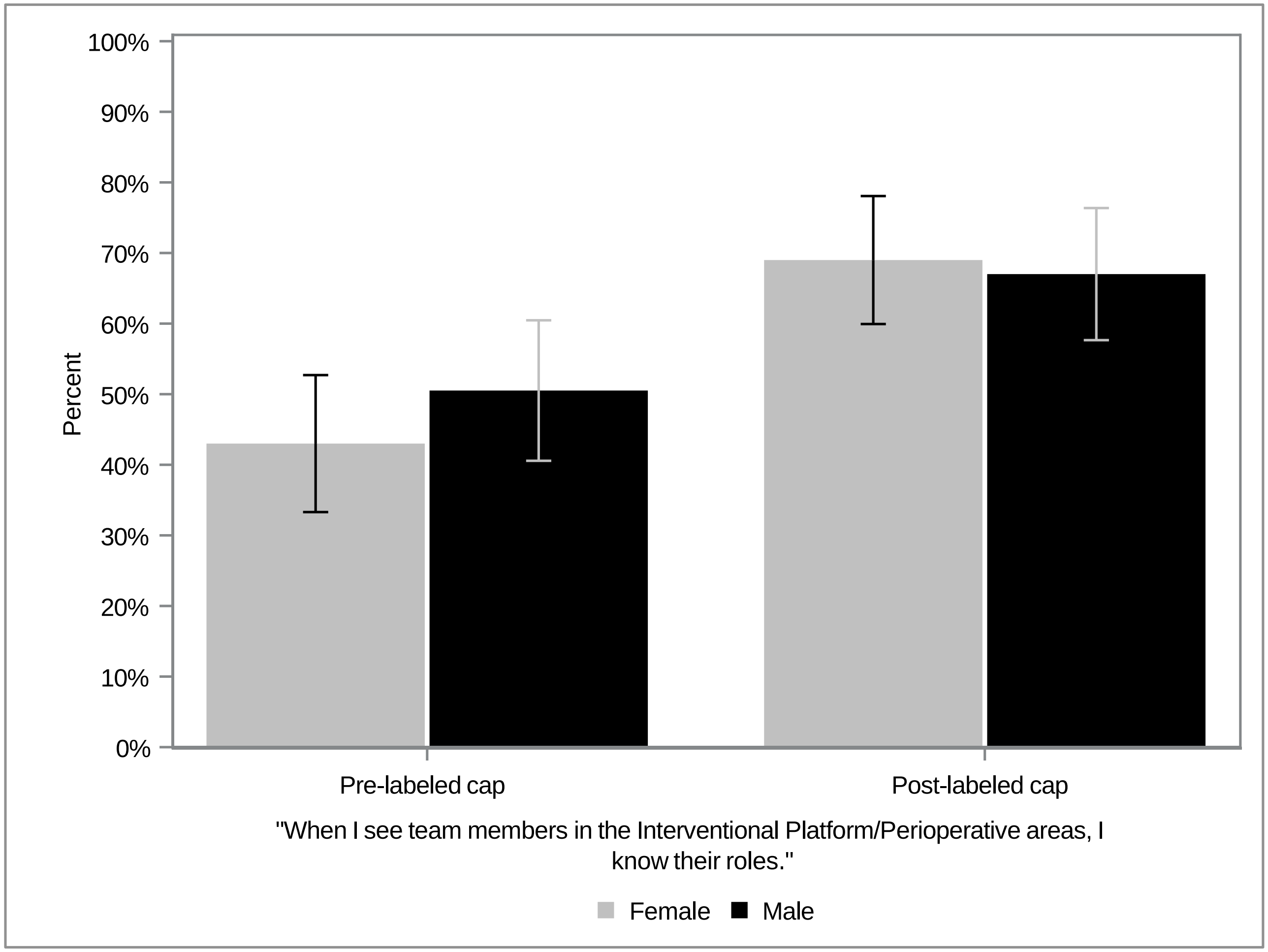

Bars represent answers of always *or* often *(vs.* sometimes, rarely*, or* never*) and are from paired responses for physicians only (N = 197). A higher percentage is favorable.*

© 2023 Wong BJ et al. *JAMA Network Open.*
