# Supplementary material for: Perceptions of Use of Names, Recognition of Roles, and Teamwork After Labeling Surgical Caps
Source: JAMA Netw Open. 2023 Nov 17;6(11):e2341182. doi: 10.1001/jamanetworkopen.2023.41182 (PMC10656635; doi:10.1001/jamanetworkopen.2023.41182)
Supplement: Supplement 2. — Data Sharing Statement [file jamanetwopen-e2341182-s002.pdf]

Data Sharing Statement

Wong. Perceptions of Use of Names, Recognition of Roles, and Teamwork After Labeling Surgical Caps. *JAMA Netw Open*. Published November 17, 2023.
doi:10.1001/jamanetworkopen.2023.41182

Data
Data available: Yes
Data types: Deidentified participant data
How to access data: mjearley@stanford.edu
When available: With publication

Supporting Documents
Document types: None

Additional Information
Who can access the data: parties whose proposed use of the data has been approved
Types of analyses: verification or expansion of project
Mechanisms of data availability: with investigator support
Any additional restrictions: Our data will be available upon request and completion of a Data Use Agreement after the study team has published all of the planned study papers.
